# The benefit of adjuvant radiotherapy on overall survival in resected stage I to II pancreatic cancer: A propensity-adjusted analysis

**DOI:** 10.1371/journal.pone.0243170

**Published:** 2020-12-14

**Authors:** Zhuang-Bin Lin, Jian-Yuan Song, An-Chuan Li, Cheng Chen, Xiao-Xue Huang, Ben-Hua Xu

**Affiliations:** 1 The Graduate School of Fujian Medical University, Fuzhou, China; 2 Department of Radiation Oncology, Fujian Medical University Union Hospital, Fuzhou, China; 3 Union Clinical Medicine College, Fujian Medical University, Fuzhou, China; 4 College of Medical Technology and Engineering, Fujian Medical University, Fuzhou, China; 5 School of Clinical Medicine, Fujian Medical University, Fuzhou, China; University of Wisconsin, UNITED STATES

## Abstract

**Background:**

The survival time of patients with early pancreatic cancer (PC) is still disappointing, even after surgical resection. PC has an extremely poor prognosis. Herein, we aimed to investigate the survival effect of postoperative radiotherapy (PORT) on resected stage I to II PC.

**Material and methods:**

A large eligible sample of patients was identified from 2010 to 2015 from the Surveillance, Epidemiology, and End Results (SEER) registry. Survival analysis was conducted to evaluate the efficiency of PORT. Propensity score matching (PSM) analysis was used to reduce selection bias and to make the groups comparable.

**Results:**

A total of 3219 patients with resected stage I to II PC was included after rigid screening. The median overall survival (OS) was 26 months with PORT (n = 1055) versus 21 months with non-PORT (n = 2164) before matching (p<0.001). By multivariable analysis, PORT remained a favorable prognostic predictor for OS. In PSM analysis, receiving PORT was associated with improved OS (median, 26 months vs. 23 months; at 2 years, 51.7% vs. 46.7%; at 5 years, 23.3% vs. 17.4% (P = 0.006). After further meticulous exploration, only the stage IIB subgroup benefited from PORT (p<0.001). This result was due to the positive lymph node state (N+), whose mortality risk was cut by 23.4% (p<0.001) by PORT.

**Conclusion:**

Addition of PORT to the treatment of patients with resected stage I to II PC conveys a survival benefit, particularly among those with N-positive or stage IIB disease.

## Introduction

Pancreatic cancer (PC) has emerged as the deadliest malignant cancer in the United States, with the lowest five-year overall survival rate of only 9%. It has become the fourth leading cause of tumor-related death and accounts for 3% of estimated new cases of cancer with an unpromising median survival of 12.4 months [1, 2]. For early-stage resected pancreatic cancer, radical surgery is the only chance for a cure and is currently the standard treatment in NCCN guidelines [3]. However, even after resection, survival remains bleak. With respect to the high risk of recurrence, adjuvant chemotherapy is recommended for those who undergo radical surgery without neoadjuvant treatment by current NCCN guidelines. The role of radiation is also being evaluated in ongoing clinical trials. Previous studies, such as the GITSG 9173 and ESPAC-1 trials, have produced controversial results, resulting in different treatment concepts between Europe and the United States [4, 5]. Even accounting for those with R1 resection and lymph node positive disease who have a higher risk for recurrence [6, 7], when considering medical costs, side effects of radiation, patient tolerance, impacts on quality of life and unsubstantiated potential benefits, the net benefit to survival from added postoperative radiotherapy (PORT) is uncertain.

Because the majority of patients are diagnosed when the disease is advanced, the percentage of those who are evaluated as “resectable” is less than 20% [8]. However, even among patients with “resectable” disease, long-term survival is only approximately 20% [5, 9, 10]. Most of the resectable designees are in the early stage. Even so, some patients with tumors evaluated as T4, which is defined by AJCC stage as unresectable, have undergone surgery with curative intent, which might be due to the experience of the doctors, the patient’s strong appeal and the development of imaging technology. To make a more persuasive case, we analyzed the impact of adjuvant radiation therapy on overall survival (OS) in resected stage I to II PC using a large publicly available cancer database.

The SEER database, established by the National Cancer Institute (NCI), provides incidence and survival data from various locations and sources throughout the United States, representing 28% of the national population. We present the latest, most precise, and most comprehensive analysis based on SEER data in resected stage I to II pancreatic cancer patients whose postoperative chemotherapy information was available to explore the role of PORT on survival in this population.

## Materials and methods

### Selecting data from SEER

We registered our account with the official SEER site and downloaded the SEER*Stat software (http://seer.cancer.gov/seerstat) to access the 1975 to 2016 SEER Research Data. The user ID is “11628-Nov2018.” Since we needed to know patients’ specific treatment information, we applied for “Radiation/Chemotherapy Databases” as well.

We used SEER*Stat software (version 8.3.6) to extract eligible cases. We selected the SEER database “Incidence-SEER 18 Regs Custom Data (with additional treatment fields), Nov 2018 Sub (1975–2016 varying)”, and selected “pancreas” for the ‘‘SEER Site Recode” variable. A limit was prescribed as “8140/3: adenocarcinoma. NOS” to the ‘‘Site and Morphology. ICD-O-3 Hist/behav” variable. Staging data were collected by limiting “Stage-7th edition. Derived AJCC Stage Group, 7th ed. (2010–2015)” to stage I to II. To avoid the invalidity of some variables of interest, only “positive histology” patients were included in the current study. In SEER, we selected “active follow-up” item, and the following three types of patients were excluded: 1) “Autopsy Only” or “Death Certificate Only”; 2) in situ cancer of the cervix uterus only; 3) not originally in active follow-up but in active follow-up now (San Francisco-Oakland only). Complete follow-up dates were also guaranteed to be available. Entries with “Autopsy only” and “Death certificate only” in the “Other. Type of reporting source” variable were excluded, as were those with “unknown” in “Cause of Death (COD) and Follow-up. Survival time.”

The exclusion criteria were as follows: (1) patients who did not receive cancer-directed surgery or did not know whether the surgery had been performed; (2) patients with unknown primary tumor site; (3) patients who had a survival <3 months; (4) patients who received other radiotherapy techniques, except external irradiation; (5) patients who did not know the sequence of radiotherapy and surgery or those who received radiation therapy prior to, both before and after, or during surgery; and (6) patients with unknown race, tumor sizes and insurance type, or any of these factors. Of note, we excluded patients who died within less than three months after diagnosis to prevent a bias for OS in the PORT arm. Those with a short OS may lose the opportunity to receive PORT, which may contribute to an unsatisfactory outcome in the non-PORT analysis arm.

### Survival analysis

All data management and statistical analyses were performed using SPSS software (version 23.0; IBM Corporation, Armonk, NY). Survival analyses were performed using the Kaplan-Meier (K-M) curve analysis method for the entire cohort. The log-rank test was used to identify differences in survival curves. Multivariate analyses calculated by Cox proportional hazard model were conducted to identify predictors of survival. Statistical significance was assumed at a threshold of P<0.05.

### Propensity score matching (PSM) analysis

Since none of the included patients were randomized into different groups, we conducted a PSM analysis, which was performed using a logistic regression model to lessen the potential selection bias between the PORT and non-PORT groups. PSM was performed based on nearest neighbor matching at a 1:1 ratio, with the value of matching tolerance as 0.0001. The current study used a PSM model to match predefined covariates, including age at diagnosis, sex, race, T stage, N stage, primary site, chemotherapy, type of insurance and type of surgery. Among them, age was matched according to the method of database collection (every five years as a period). Survival analysis was conducted in the matched population.

## Results

**Fig 1:**
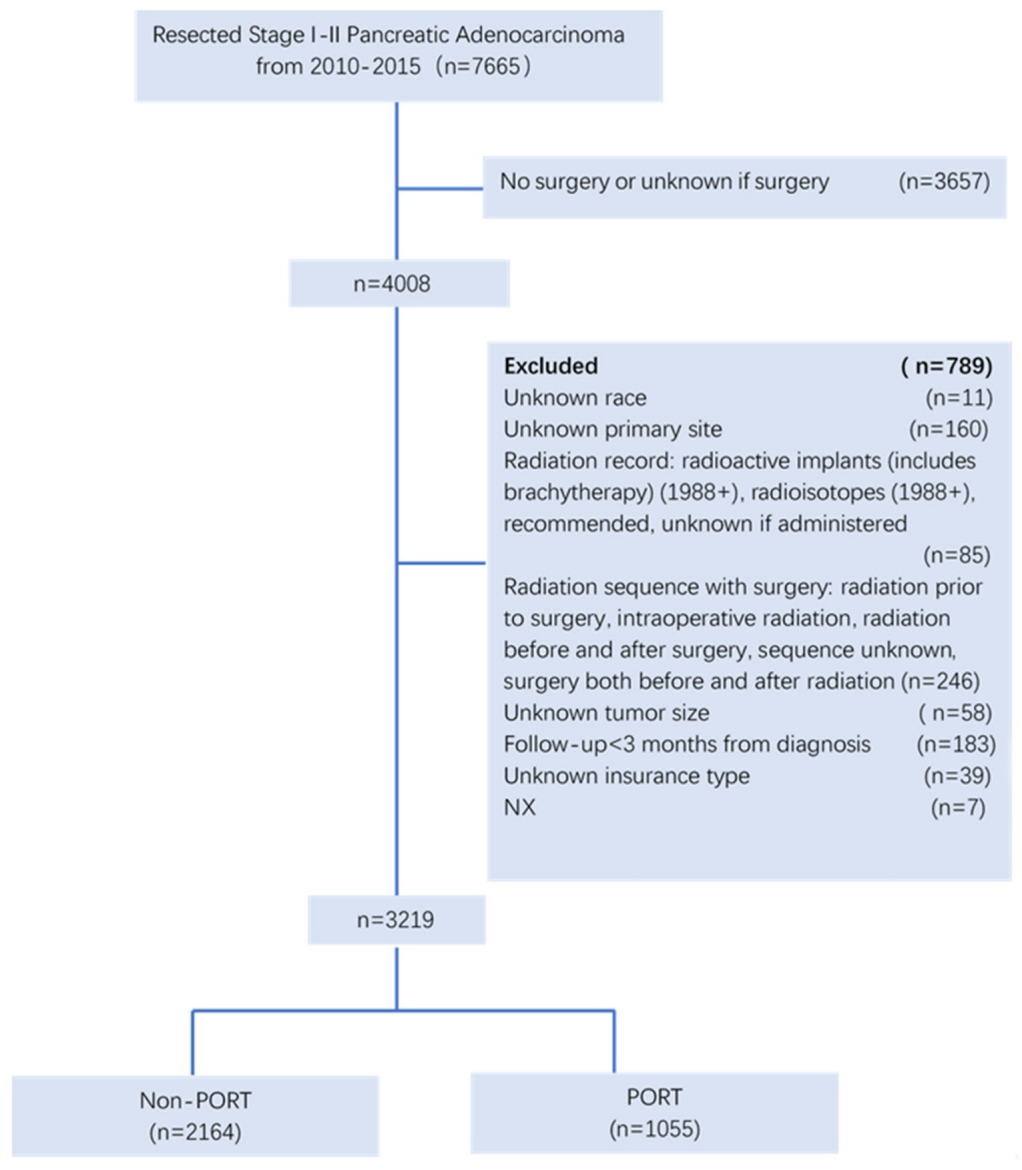
Patient selection diagram.

We identified 7665 resected stage I to II pancreatic cancer patients from the SEER database who met the inclusion criteria (selection diagram illustrated in Fig 1); 3219 patients with complete clinical information were ultimately included in this study. All 3219 patients in this study were diagnosed during 2010–2015. Baseline characteristics are listed in Table 1. The majority of patients were white (82.9%) and insured (89.0%), had stage II (90.1%), T3 (83.6%), and node-positive (65.2%) disease with a tumor size<5 cm (87.4%), and had primary disease located in the pancreatic head (77.7%). Utilization of chemotherapy (CT) occurred in 25.2% of patients. More than 60% in this cohort did not receive PORT (67.2%). Local or partial pancreatectomy was performed in the majority of patients (81.4%).

**Table 1:** Characteristics of stage I to II pancreatic adenocarcinoma patients who underwent surgical resection (n = 3219).

Characteristic	Patients (%)	Overall survival (OS)	
		Median (mo)	P-value
**Race**			0.651
Black	315 (9.8)	22	
White	2669 (82.9)	22	
Other	235 (7.3)	23	
**Age (years)**			<0.001
<70	1897 (58.9)	25	
≥70	1322 (41.1)	19	
**Sex**			0.022
Male	1654 (51.4)	21	
Female	1565 (48.6)	23	
**Primary site**			0.133
Head	2502 (77.7)	22	
Body	226 (7.0)	26	
Tail	311 (9.7)	23	
Overlapping lesion	115 (3.6)	21	
Duct	28 (0.9)	20	
Other specified parts	37 (1.1)	26	
**AJCC stage (7th)**			<0.001
IA	129 (4.0)	69	
IB	190 (5.9)	34	
IIA	802 (24.9)	27	
IIB	2098 (65.2)	20	
**T stage**			<0.001
T1	176 (5.5)	46	
T2	352 (10.9)	28	
T3	2691 (83.6)	21	
**N stage**			<0.001
N0	1121 (34.8)	30	
N1	2098 (65.2)	20	
**Type of surgery**			0.332
Local or partial pancreatectomy	2619 (81.4)	22	
Total pancreatectomy	388 (12.1)	21	
Extended pancreatoduodenectomy	164 (5.1)	22	
Surgery/pancreatectomy, NOS	48 (1.5)	18	
**PORT**			<0.001
No	2164 (67.2)	21	
Yes	1055 (32.8)	26	
**Chemotherapy**			<0.001
Yes	812 (25.2)	15	
No	2407 (74.8)	24	
**Tumor size (cm)**			<0.001
<5	2814 (87.4)	23	
≥5	405 (12.6)	17	
**Insurance recode**			<0.001
Uninsured	81 (2.5)	25	
Insured	2864 (89)	22	
Any Medicaid	274 (8.5)	19	

### Survival analysis before PSM

**Fig 2:**
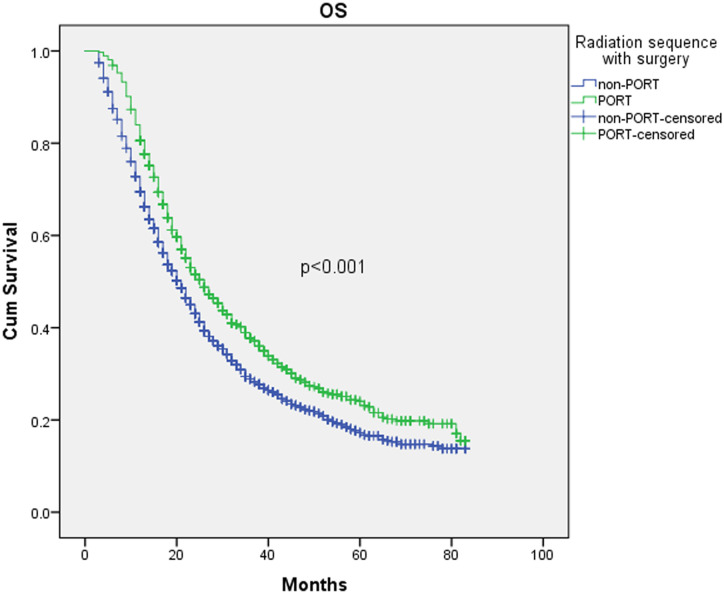
Kaplan–Meier analysis of overall survival of PORT (n = 1055) and non-PORT (n = 2164) groups before PSM.

The included population in the study numbered 3219 before PSM. There were 1008 patients surviving at the end of follow-up. The median, two-year, and five-year OS was 22 months (95% CI 21.0–23.0), 45.9% (95% CI 44.1%-47.7%), and 19.6% (95% CI 17.8%-21.4%), respectively. Fig 2 shows the survival curves according to receipt of PORT by K-M analysis. It was readily observed that patients receiving PORT (n = 1055) versus non-PORT (n = 2164) had significantly improved OS. The OS rates at two and five years increased by 8% and 7%, respectively. Median OS was 26 months (95% CI 23.8–28.2) with PORT versus 21 months (95% CI 19.8–22.1) with non-PORT, P<0.001.

Our K-M analysis results also revealed that older age, male sex, high AJCC stage, lymph node involvement, lack of chemotherapy, larger tumor size, and Medicaid benefits were associated with worse OS, as shown in Table 1. These aforementioned factors proved to be adverse prognostic factors for OS after Cox multivariable analyses, as shown in Table 2.

**Table 2:** Cox analysis of the unmatched population for OS.

	HR	95% CI	Sig.
**Age (years)**			
<70	-	-	-
≥70	1.246	1.142–1.359	<0.001
**Sex**			
Male	-	-	-
Female	0.895	0.823–0.973	0.01
**PORT**			
No	-	-	-
Yes	0.824	0.747–0.909	<0.001
**Chemotherapy**			
No	-	-	-
Yes	0.652	0.589–0.723	<0.001
**Tumor size (cm)**			
<5	-	-	-
≥5	1.267	1.122–1.432	<0.001
**Insurance recode**			<0.001
Uninsured	-	-	-
Insured	1.417	1.051–1.910	0.022
Any Medicaid	1.888	1.366–2.610	<0.001
**T stage**			<0.001
T1	-	-	-
T2	1.834	1.417–2.373	<0.001
T3	2.134	1.693–2.689	<0.001
**N stage**			
N0	-	-	-
N1	1.596	1.451–1.755	<0.001

### Survival analysis after PSM

The PSM process achieved a balance between the two groups (n = 782 for each group). Details of the covariates in the groups after PSM are shown in Table 3. The two groups were well matched, depending on the small and appropriate match tolerance. The table also indicates minimization of potential confounders between the two groups.

**Table 3:** Patient characteristics after PSM.

Characteristics	Patients (%)	
	Non-PORT (N = 782)	PORT (N = 782)
**Race**		
Black	55 (7.0)	64 (8.2)
White	690 (88.2)	683 (87.3)
Other	37 (4.7)	35 (4.5)
**Age (years)**		
<70	520 (66.5)	520 (66.5)
≥70	262 (33.5)	262 (33.5)
**Sex**		
Male	423 (54.1)	413 (52.8)
Female	359 (45.9)	369 (47.2)
**Primary site**		
Head	675 (86.3)	672 (85.9)
Body	36 (4.6)	39 (5.0)
Tail	50 (6.4)	49 (6.3)
Overlapping lesion	13 (1.7)	14 (1.8)
duct	3 (0.4)	5 (0.6)
Other specified parts	5 (0.6)	3 (0.4)
**AJCC stage(7th)**		
IA	9 (1.2)	9 (1.2)
IB	34 (4.3)	27 (3.5)
IIA	180 (23.0)	186 (23.8)
IIB	559 (71.5)	560 (71.6)
**T stage**		
T1	16 (2.0)	14 (1.8)
T2	62 (7.9)	59 (7.5)
T3	704 (90.0)	709 (90.7)
**N stage**		
N0	223 (28.5)	222 (28.4)
N1	559 (71.5)	560 (71.4)
**Type of surgery**		
Local or partial pancreatectomy	658 (84.1)	671 (85.8)
Total pancreatectomy	91 (11.6)	79 (10.1)
Extended pancreatoduodenectomy	25 (3.2)	26 (3.3)
Surgery/pancreatectomy, NOS	8(1.0)	6 (0.8)
**Chemotherapy**		
Yes	755 (96.5)	755 (96.5)
No	27 (3.5)	27 (3.5)
**Tumor size (cm)**		
<5	684 (87.5)	688 (88.0)
≥5	98 (12.5)	94 (12)
**Insurance recode**		
Uninsured	7 (0.9)	11 (1.4)
Insured	735 (94.0)	734 (93.9)
Any Medicaid	40 (5.1)	37 (4.7)

**Fig 3:**
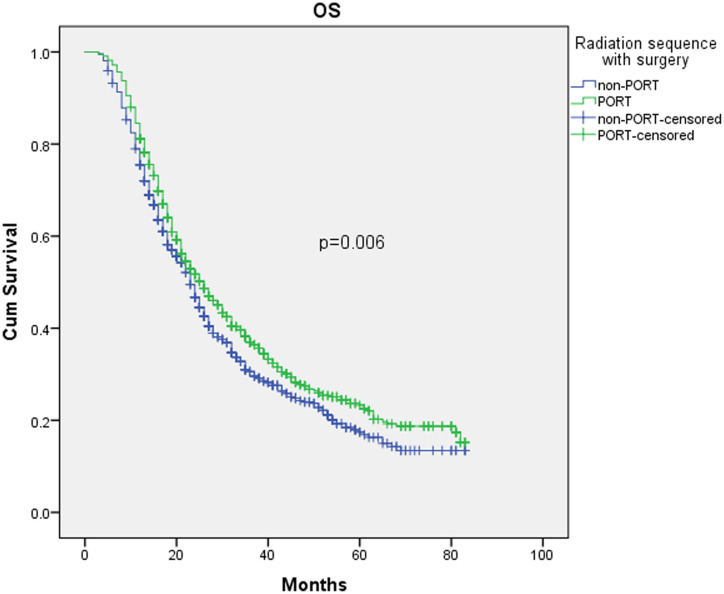
Kaplan–Meier analysis of overall survival of PORT (n = 715) and non-PORT (n = 715) groups after PSM.

Though the OS curves became closer after PSM (as shown in Fig 3), the difference in survival between the two groups remained significant (P = 0.006). The median, two-year, and five-year OS was 26 months (95% CI 23.5–28.5), 51.7% (95% CI 48.2%-55.2%), and 23.3% (95% CI 19.6%-27.0%) for the PORT (n = 782) group versus 23 months (95% CI 21.3–24.7), 46.7% (95% CI 43.0%-50.4%), and 17.4% (95% CI 13.7%-21.1%) for the non-PORT (n = 782) group, respectively.

The results of K-M analysis for OS are listed in Table 4. Shorter median OS was significantly associated with older age, later AJCC stage, high-level T stage, lymph node involvement, no PORT or chemotherapy, larger tumor size and receipt of any Medicaid. In the multivariate Cox regression analysis shown in Table 5, age, PORT, chemotherapy, tumor size, lymph node state and type of insurance were independent prognostic factors of OS.

**Table 4:** K-M analysis of the matched population for OS.

Characteristics	Patients (%)	Overall survival (OS)	
		Median (mo)	P value
**Race**			0.611
Black	119 (7.6)	26	
White	1373 (87.8)	24	
Other	72 (4.6)	18	
**Age (years)**			0.035
<70	1040 (66.5)	26	
≥70	524 (33.5)	21	
**Sex**			0.253
Male	836 (53.5)	24	
Female	728 (46.5)	25	
**Primary Site**			0.998
Head	1347 (86.1)	24	
Body	75 (4.8)	24	
Tail	99 (6.3)	23	
Overlapping lesion	27 (1.7)	23	
duct	8 (0.5)	14	
Other specified parts	8 (0.5)	20	
**AJCC Stage (7th)**			<0.001
IA	18 (1.2)	31	
IB	61 (3.9)	34	
IIA	366 (23.4)	32	
IIB	1119 (71.5)	32	
**T stage**			0.045
T1	30 (1.9)	31	
T2	121 (7.7)	28	
T3	1413 (90.3)	23	
**N stage**			<0.001
N0	445 (28.5)	32	
N1	1119 (71.5)	22	
**Type of surgery**			0.641
Local or partial pancreatectomy	1329 (85.0)	24	
Total pancreatectomy	170 (10.9)	23	
Extended pancreatoduodenectomy	51 (3.3)	26	
Surgery/pancreatectomy, NOS	14 (0.9)	30	
**PORT**			0.006
No	782 (50.0)	23	
Yes	782 (50.0)	26	
**Chemotherapy**			0.037
Yes	1510 (96.5)	24	
No	54 (3.5)	17	
**Tumor size (cm)**			0.015
<5	1372 (87.7)	25	
≥5	192 (12.3)	20	
**Insurance recode**			0.035
Uninsured	18 (1.2)	24	
Insured	1469 (93.9)	24	
Any Medicaid	77 (4.9)	20	

**Table 5:** Cox analysis of the matched population for OS.

	HR	95% CI	Sig.
**Age (years)**			
<70	-	-	-
≥70	1.173	1.034–1.332	0.013
**Sex**			
Male	-	-	-
Female	0.895	0.823–0.973	0.01
**PORT**			
No	-	-	-
Yes	0.835	0.740–0.942	0.003
**Chemotherapy**			
No	-	-	-
Yes	0.703	0.513–0.963	0.028
**Tumor size (cm)**			
<5	-	-	-
≥5	1.262	1.055–1.509	0.011
**Insurance recode**			0.016
Uninsured	-	-	-
Insured	0.929	0.523–1.649	0.801
Any Medicaid	1.402	0.752–2.612	0.287
**N stage**			
N0	-	-	-
N1	1.583	1.374–1.823	<0.001

### The benefit of PORT in different stages

**Fig 4:**
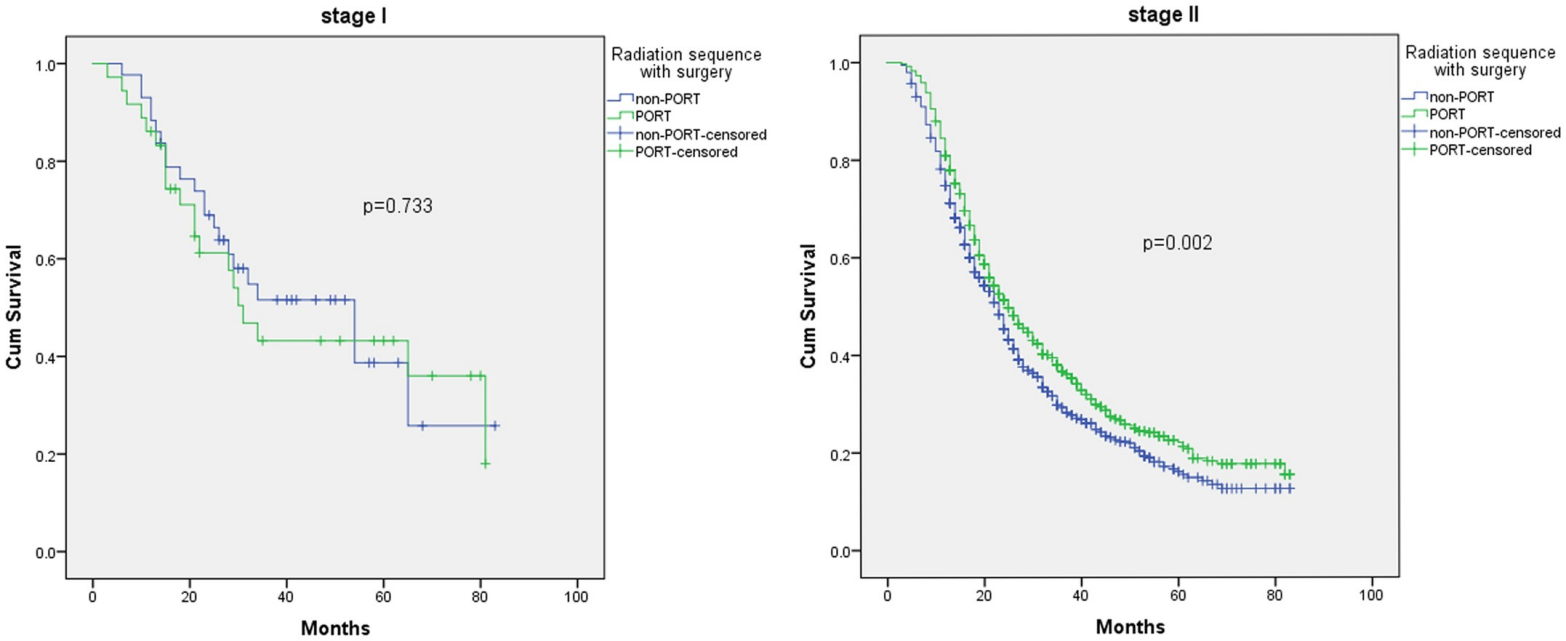
K-M analysis of overall survival of PORT and non-PORT in different AJCC stage groups.

Fig 4 shows the OS curves in K-M analysis of PORT in different AJCC stage subgroups. We found that there was no significant difference between stage I (P = 0.733) subgroups. For the stage I group, K-M analysis for OS showed that there was no factor associated with poor outcome, and there was no significant prognostic factor for OS after multivariate Cox analysis.

However, the median OS values for PORT (n = 746) and non-PORT (n = 739) in stage II subgroups were 25 months (95% CI 22.3–27.7) and 23 months (95% CI 21.2–24.8) (P = 0.002), respectively. Furthermore, poor OS was significantly associated with higher N stage (p<0.001), no chemotherapy (p<0.001), larger tumor size (P = 0.006), older age (P = 0.028) and type of insurance (P = 0.009) after K-M analysis. Older age (P = 0.017, HR:1.171, 95% CI 1.029–1.333), receiving PORT (P = 0.002, HR:0.824, 95% CI 0.728–0.931) and chemotherapy (P = 0.008, HR:0.648, 95% CI 0.471–0.892), higher N stage (p<0.001, HR:1.515, 95% CI 1.303–1.763), larger tumor size (P = 0.005, HR:1.294, 95% CI 1.079–1.551) and type of insurance (P = 0.009) remained prognostic factors for OS in the multivariate Cox regression analysis.

### The benefit of PORT in different N-stage

**Fig 5:**
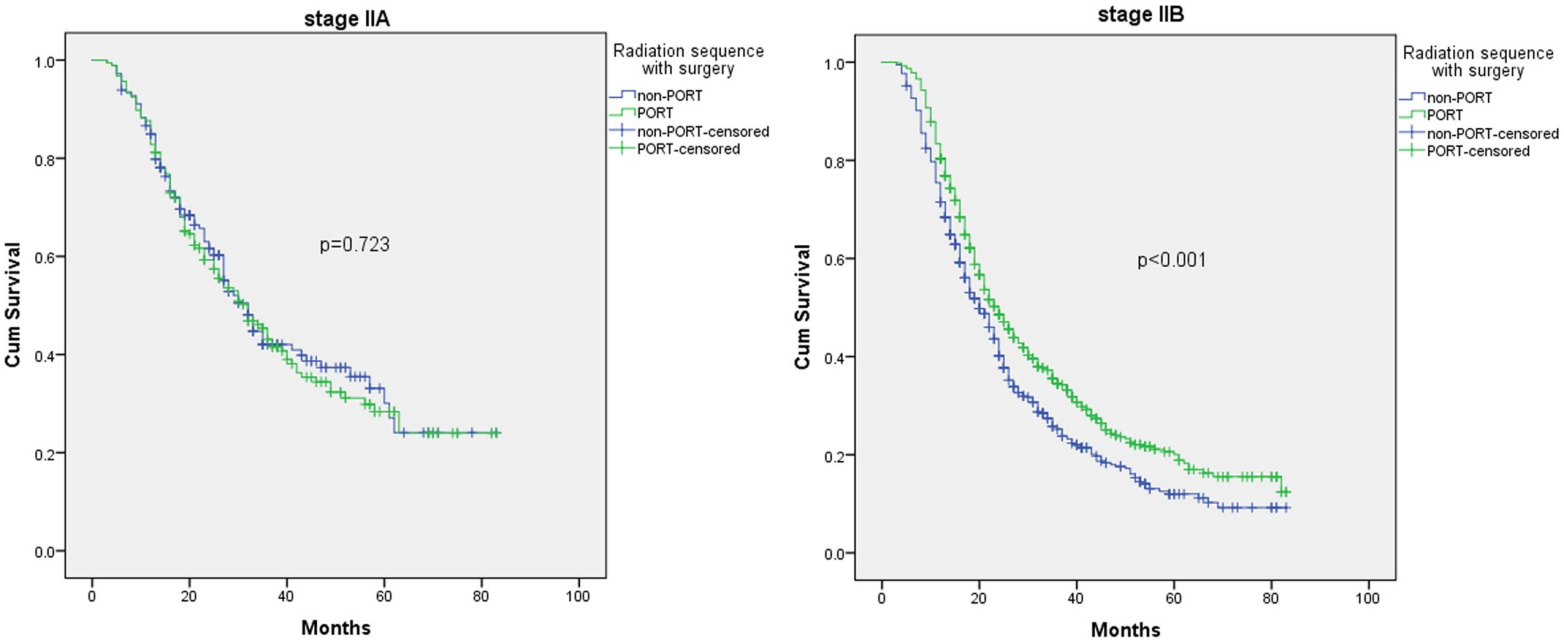
K-M analysis of overall survival of PORT and non-PORT in stage IIA and IIB.

To elucidate the underlying factors that are sensitive to PORT among stage II patients, we meticulously analyzed subgroups of stage IIA and stage IIB first. As shown in Fig 5, we found that only stage IIB is associated with an OS benefit from PORT (p<0.001).

**Fig 6:**
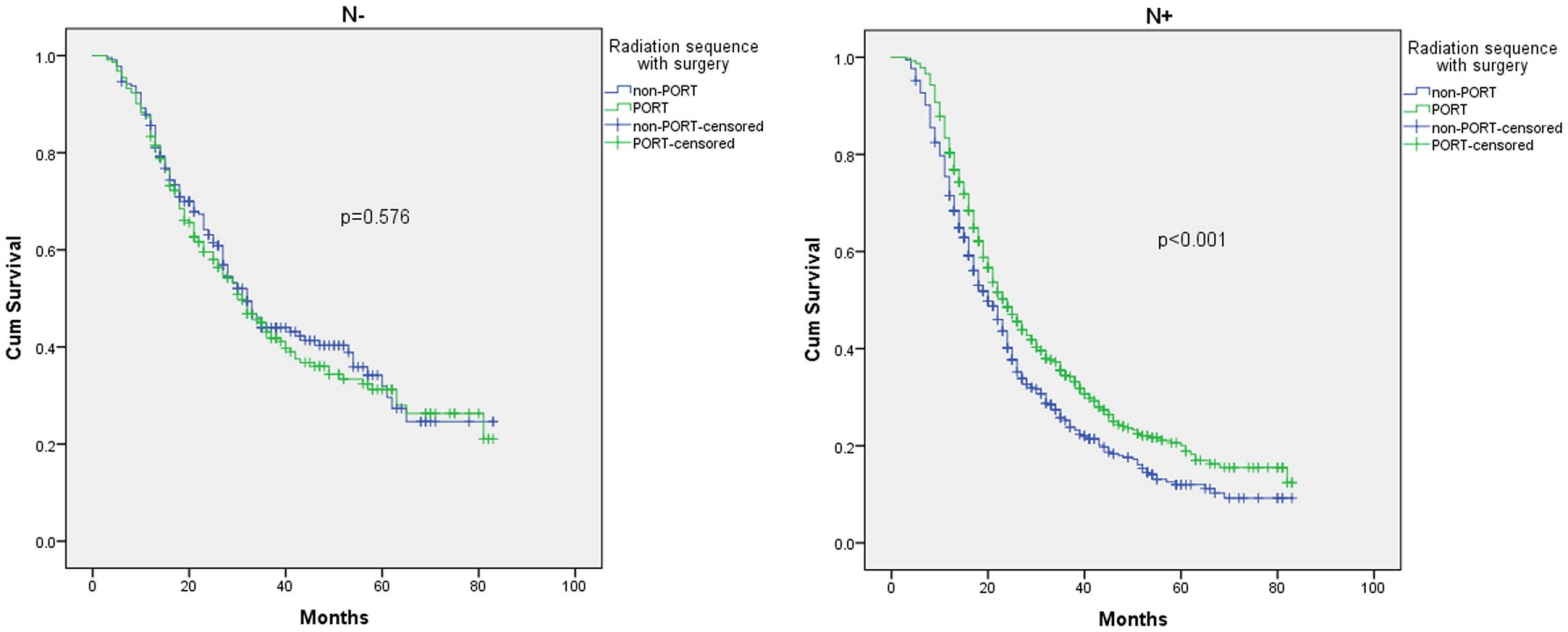
K-M analysis of overall survival of PORT and non-PORT in different N-stage groups.

The most obvious difference between stage IIA and stage IIB is whether there is lymph node involvement. Given the previous result of stage I, we considered that lymph node state may play a key role in patient prognosis, and further research is to be conducted. To investigate whether N-status contributes to an improved OS in stage IIB patients, we performed further analyses, and the final result is shown in Fig 6. We found that only those with lymph node involvement obtained an OS benefit from PORT with a 23.4% reduction in the risk of death (p<0.001). For N- group, the K-M analysis for OS showed that poor outcome was significantly associated with larger tumor size (P = 0.047). In the multivariate Cox regression analysis, larger tumor size (P = 0.038, HR:1.442, 95% CI 1.020–2.037) and older age (P = 0.047, HR:1.290, 95% CI 1.003–1.658) were adverse prognostic factors for OS. For the N+ group, K-M analysis for OS showed that poor outcome was significantly associated with no PORT (p<0.001) and type of insurance (p<0.001). In addition, receiving PORT (p<0.001, HR:0.766 95% CI 0.667–0.879) and type of insurance (p<0.001) remained prognostic factors for OS in the multivariate Cox regression analysis.

## Discussion

SEER is a large, complex, multi-institutional database that affords many advantages to researchers. In this study, we used SEER to identify an eligible sample of patients with resected early-stage (I to II) pancreatic cancer in an attempt to examine the benefit of adding PORT to the treatment protocol for this population. We stratified our analysis to allow greater depth to identify more clearly which subgroups might benefit from PORT. Due to the shortcomings of the database, however, we had to utilize appropriate statistical means to minimize the resulting impacts.

Results demonstrated that addition of PORT was associated with an OS benefit in resected stage I to II PC on the whole. To produce more sound results and make the two groups meaningfully comparable, PSM was performed. Our outcome of preferable OS among patients with PORT versus non-PORT remained significant after PSM. We found that only stage IIB benefited from PORT, which prompted us to query whether lymph node involvement (N+ subgroup) helped explain this result. Subsequent research confirmed the hypothesis that only the N+ group acquired longer OS after PORT.

Considering the conflicting results of previous randomized clinical trials, the role of PORT in resected pancreatic cancer remains debatable [4, 11]. The potential of PORT to help patients obtain better OS was first published by the Gastrointestinal Tumor Study Group (GITSG) 9173 study in the 1980s [4]. That study included 43 patients with negative-margin (R0) resection. Patients were randomized to observation (n = 22) or combined therapy (n = 21). The postoperative chemoradiotherapy (CRT) regimen was well tolerated due to the absence of any life-threatening side effects or toxic reactions. Median survival for the adjuvant CRT group was significantly longer than that observed for the surgery-only group (20 months vs. 11 months, P = 0.03). Although many researchers criticized the study for its weaknesses, adjuvant CRT is encouraged for typical treatment of early-stage resectable patients in the United States, which is an interesting result. The study was limited to patients who underwent margin-negative resection but were in slow disease progression, which created a selection bias that made the results less persuasive. Since the PORT regimen was not examined in isolation, the survival benefits may have come from the effects of systematic chemotherapy (CT). It was also limited by small sample size, outdated radiotherapy regimens, and exclusion of lymph node-negative patients. Later, the European Organization for the Research and Treatment of Cancer (EORTC) 40891 study had a similar design to GITSG 9173 [12], yet achieved the exact opposite result. EORTC randomized 218 resected patients to either 5-FU-based CRT or observation and found equivalent survival outcomes between the two groups. This trial was limited by poor compliance with chemotherapy protocol in the experimental arm. In addition, 541 resected patients in the ESPAC-1 trial were randomized to surgery-only or adjuvant treatment [5]. The options for neoadjuvant therapy included CRT alone, CT alone and CRT followed by CT. The study concluded that adding radiotherapy did not improve OS. There were some limitations contributing to the undesirable result, including the high rate of non-adherence, the inadequate radiation techniques (lack of quality assurance, split course regimens, nonstandard RT dose) and the majority of suboptimal patients in the CRT arm. The EORTC-40013/FFCD-9203/GERCOR phase II study evaluated adjuvant CT followed by CRT versus CT only [11]. Median OS was 24 months in both groups, and no benefit to OS was observed. However, CRT reduced the local recurrence rate (11% vs. 24%) and was well tolerated.

After further subgroup analysis, PORT was fortunately found to reduce the risk of death in lymph node-positive patients by 23.4%. Lymph nodal status is the key to distinguishing prognosis of different stages. There was no significant benefit to OS in PORT group versus non-PORT group in either stage I (T1-2N0M0) or stage IIA (T3N0M0). Only patients evaluated as stage IIB (node-positive) achieved better survival from PORT. Current clinical trials had insufficient power to look into the survival benefit in patients with node-positive disease. However, prior analyses based on shared databases have reported results similar to ours. Moody et al. reviewed the SEER data (1988–2003) and demonstrated that only resected stage IIB disease (T1-T3N1M0) obtained better OS from PORT [13]. Unfortunately, that study falls short in that the survival benefit derived at least in part from various forms of CT, yet the database is not replete with detailed CT data. Another analysis of NCDB data (1998–2009 dataset) conducted by Rutter et al. also examined the addition of radiotherapy to adjuvant CT. That protocol was significantly associated with longer OS among patients with pT3 (p = 0.003) or pN1 (p<0.001) disease [14]. The benefit may primarily rely on local control. Surgical resection is the only treatment with curative potential for pancreatic cancer. However, median survival decreases to 19 months or less when lymph nodes are involved [15]. A previous study demonstrated that PORT increased the local control rate in resected patients with node-positive by 20% [16]. However, the late effects of radiotherapy are still worthy of attention.

There have been many prior studies of the SEER database reporting survival benefits from PORT for the resected population [13, 17–24]. However, nearly all of the early studies lack data on chemotherapy, and it is unclear whether any OS benefit is gained by systematic therapy.

The National Cancer Data Base (NCDB) in the United States is another large population-based database. There is a certain amount of NCDB research about adjuvant radiotherapy in resected pancreatic cancer [14, 25–27]. Kooby et al. used the NCDB to analyze the outcome of adjuvant therapy on OS in pancreatic adenocarcinoma [25]. Their results showed that CRT obtained the greatest benefit to OS (HR 0.70, 95% CI 0.61–0.80) when compared to chemotherapy only (HR 1.04, 95% CI 0.93–1.18) and non-adjuvant. Rutter et al. analyzed the impact of PORT in the pT1-3N0-1M0 population using PSM [14]. That study demonstrated that adjuvant CRT was strongly associated with superior OS among patients who underwent R1 resection (P = 0.003) or who were evaluated as having pN1 disease (P = 0.03). Kantor et al. collected cases with stage I to II PC using NCDB (2004–2012) [26]. All included patients underwent upfront margin-negative resection. For patients who underwent R0 resection, the study showed that adding radiation to postoperative chemotherapy was a better choice in terms of adjuvant regimen. Ostapoff et al. analyzed stage I resected patients, who comprise less than 15% of the population (2006–2012) [8]. Their results showed that stage IA patients experienced a decrease in OS from adding PORT to adjuvant chemotherapy, while no such association was observed in stage IB patients.

In many studies supported by large-repository data, PSM is used to solve endogenous population problems and to create highly comparable comparison groups. The current study adopted this statistical method to minimize treatment selection bias. Although there have been many similar encouraging studies providing valuable information, the present study has several design advantages. First, this study performed PSM at a ratio of 1:1, which significantly increases the efficacy of the research. Second, we limited the population to patients with stage I to II resected PC, which eliminates the effects of the T4 subgroup, which involves a vital artery. Third, the distribution of baseline characteristics before and after matching was clearly compared in tabular form, demonstrating that all baseline characteristics of each subgroup were well balanced after PSM. Fourth, through stratified analysis, we delineated which groups benefit from PORT. Our study shows that only N-positive patients obtain benefits from PORT, which is consistent with conclusions from other studies [13, 14, 26]. Finally, we assessed chemotherapy data through special requests, which had not been done in earlier SEER studies.

Although the SEER database is a powerful tool to briefly characterize national trends and collect detailed data such as pathologic or treatment information that takes advantage of a large patient population, there are still some limitations to this study. It is confounding that the SEER database fails to offer complete and detailed information of therapy experience (the types, schemes and timing of chemotherapy or radiotherapy, etc.). Furthermore, the prognosis for different PORT strategies varies widely. These factors, regarded as potential pitfalls, might alter the results of the study. In addition, information on variables, such as operative margins and patient functional status, which play an important role in OS outcomes, was not collected in the SEER database and could influence postoperative treatment selection and introduce treatment bias. Fortunately, given our strict access principles, we feel that this study, based on our dataset design, represents a very valid and persuasive result.

## Conclusions

Although PORT is associated with improved OS for stage I to II patients as a whole, we observed that the trend appears to be generated by stage IIB or N-positive resected patients in a large national database using PSM. Most patients, especially those with pancreatic head cancer, significantly benefit from PORT. These data merit consideration and the final results of ongoing RTOG 0848 clinical trial are eagerly awaited. We believe that PORT will be accepted by more patients and clinicians in the future.
